# Passive Heating: Reviewing Practical Heat Acclimation Strategies for Endurance Athletes

**DOI:** 10.3389/fphys.2018.01851

**Published:** 2018-12-20

**Authors:** Storme L. Heathcote, Peter Hassmén, Shi Zhou, Christopher J. Stevens

**Affiliations:** ^1^School of Health and Human Sciences, Southern Cross University, Lismore, NSW, Australia; ^2^Centre for Athlete Development, Experience & Performance, Southern Cross University, Coffs Harbour, NSW, Australia

**Keywords:** passive heating, heat acclimation, hot-water immersion, thermal adaptation, hot bath, sauna, athletes

## Abstract

Heat acclimation protocols—both active and passive—have been employed by athletes in an effort to attenuate the detrimental effects of heat stress on physical capacities and sports performance. Active strategies have been extensively reviewed, but have various practical and economic limitations. The purpose of this review was therefore to provide an overview of the passive strategies that have received less attention, yet may be more practical or economically viable; recommendations for athletes are also provided. With a systematic search of the relevant databases ending in June 2018, 16 articles on passive heat acclimation that met the inclusion criteria were included in the review. The review highlighted that passive heat acclimation strategies can successfully induce heat adaptations, evident by reports of improved exercise performance, thermoregulatory, cardiovascular, and perceptual responses accompanying such interventions. Based on the review it is apparent that the use of sauna, hot-water immersion and environmental chambers may be used to provide heat stress under passive conditions, for the purpose of acclimation. To maximize the thermoregulatory-adaptive responses, exercise bouts should be employed prior to passive heat stress, rather than passive heating alone, with a minimal delay between exercise and the application of heat stress. Heating bouts should have a minimum duration of 30 min per session and be employed on consecutive days, when possible, with a minimum of 6–7 exposures to induce adaptation. This review identified that information regarding the magnitude of performance changes that can occur, as well as the perceptual responses to passive heating protocols is limited. Future research should investigate the use of passive heat exposures before and/or after repeated heat training sessions, to assess if a further boost to heat adaptation can be achieved with this strategy.

## Introduction

A hot (30–40°C) and humid (>60%) environment can pose a significant thermoregulatory and perceptual challenge for an endurance athlete, while also elevating their risk of experiencing heat illnesses, especially in individuals unaccustomed to such conditions (Casadio et al., 2016; Racinais et al., 2017). Athletes may also be at increased risk of dehydration when performing in the heat due to the thermoregulatory adjustments that allow for increased heat dissipation, such as enhanced sweat rate and skin blood flow (Racinais et al., 2015). As such, exercise performance may be compromised when conducted in hot ambient conditions, while heat acclimatization (exposure to natural outdoor heat) or acclimation training (exposure to simulated environmental heat; both abbreviated to HA) can attenuate the performance decrements (Costa et al., 2014; Racinais et al., 2015). Effective HA protocols result in numerous physiological adaptations, e.g., decreasing exercising and resting core temperature and heart rate, plasma volume expansion, earlier onset of sweating and higher sweat output. These adaptations contribute to improvements in cardiovascular stability, sweating capacity and thermoregulation, processes that work in combination to improve physical capacities and sports performance in the heat (Garrett et al., 2011; Costa et al., 2014; Périard et al., 2015). To date, HA has been achieved via both active strategies, using various modes of exercise outdoors or in a heat chamber (Lorenzo et al., 2010), and passive strategies which do not require exercise in the heat (Zurawlew et al., 2018).

There are three most commonly used active strategies explored within the literature, including (1) thermal clamping, commonly referred to as the “controlled hyperthermia technique,” where a target core temperature is maintained; (2) exercising at a constant predetermined workload in hot and/or humid conditions, referred to as the “constant work rate technique”; and (3) the “self-regulated technique,” where the individual selects their own work rate during exercise heat exposure (Taylor, 2006; Périard et al., 2015). The duration of these protocols has been categorized as short term (< 7 days), medium term (8–14 days) and long term (>14 days) (Garrett et al., 2011). Short term protocols are more convenient for athletes with heavy training regimes (Zurawlew et al., 2016), while medium term protocols elicit greater physiological adaptations and performance improvements (Guy et al., 2015). However, the long term regimes offer the greatest adaptations (Tyler et al., 2016). Despite the absence of a clear consensus on the precise session duration per heat exposure (Daanen et al., 2018), current recommendations state that in order to achieve the physiological and performance benefits, athletes should train on consecutive days, across 1–2 weeks and begin preparations at least 2-weeks before the major event prior to tapering (Périard et al., 2015; Racinais et al., 2015; Tyler et al., 2016; Daanen et al., 2018). Furthermore, protocols can be made more effective by using the same exercise mode as employed in competition, mimicking competition ambient temperatures, ensuring specific humidity exposure, considering individual differences in athletes' responses to HA, as well as adjusting intensity according to the training phase and time available to the individual (Taylor, 2006; Guy et al., 2015; Casadio et al., 2016; Tyler et al., 2016).

The active strategies have been widely used and extensively reviewed in the literature (Tyler et al., 2016). For athletes residing in cool/temperate environments, the use of these active protocols would require travel to a hot outdoor environment or exercise in a heat chamber, which may be logistically difficult and impractical (Casadio et al., 2016). Such methods may also interfere with both normal training and/or specific taper sessions, perhaps making them dangerous to an athlete's training outcome. In contrast, the use of passive strategies may be more practical and cost effective, especially for those living in a cool climate. The main stimulus for individuals to experience heat adaptation, is repeated episodes of elevated core temperature, therefore passive HA interventions may present an effective alternative to traditional active-based strategies, since repeated physical exercise in the heat may not be necessary for HA (Regan et al., 1996; Brazaitis and Skurvydas, 2010). This review will summarize the current passive heating literature, including performance, physiological and perceptual adaptations that can be induced, and provide recommendations for athletes.

## Passive Heat Acclimation Strategies

### Literature Search Methods and Retrieval

This review was based on searches conducted in relevant databases (Medline, Science Direct Freedom Collection, Highwire Press American Physiological Society, SAGE journals, ProQuest Science Journals, SPORTDiscus and Google Scholar) ending in June 2018, in the areas of HA strategies and the various methods that have been used/ tested, the resultant benefits for athletes and the responses that occur following HA. Database search terms included “heat acclimation,” “heat-acclimatization,” “thermal adaptation,” “hot-water immersion,” “hot bath,” “sauna,” “heat chamber,” “exercise,” “performance,” “perception,” “heat stress,” and their various truncations. Inclusion criteria stipulated that articles were written in English and investigated human subjects. A total of 16 articles met these criteria, which are summarized in Table 1.

**Table 1:** Summary of the performance, physiological and perceptual responses to passive strategies for heat acclimation.

**Investigation**	**Environmental conditions**	**Subjects**	**Heat acclimation method**	**Performance response**	**Physiological and perceptual responses**
Stanley et al., 2015	Not reported	Number: 7 (no CN) Sex: Male Training status: well-trained cyclists	Post-exercise sauna bathing (30 min) Conditions: 87°C, 11% RH (sauna) Duration: 35 days.	↔ Peak PO in a graded cycling test, starting at 175 W, increasing by 25 W/min	↑PV and HR_(ex)_ ↓HRR_60s_ ↔ HR_(Peak)_ ↓HR_(wake)_ (Trivial to moderate)
Scoon et al., 2007	Not reported	Number: 6 (same group was CN) Sex: Male Training status: competitive distance runners/triathletes	Post-running sauna bathing (31 min) Conditions: 89.9°C (sauna) (12 sessions) Duration: 21 d (alternate).	↑TTE in a treadmill run at constant speed (runner's best speed over 5 km) (+32%)	↑PV, RCV, BM and TBV
Leppaluoto et al., 1986	Not reported	Number: 10 Sex: Male	Finnish sauna dry heat exposure (60min) twice daily Conditions: 80°C (sauna) Duration: 7 d.		↓HR_(rest)_ and T_re(rest)_ ↓Serum K, Na and Fe ↔ ECG recordings
Shido et al., 1999	Exp1: 27°C, 50% RH (24 h.) Exp2: 28°C, 40% RH (1 h), 42.0°C HWI after 30 min (legs) repeated pm.	Number: 6 in each Exp Sex: Male and Female	Seated rest in HC (4 h) Conditions: 46.0°C, 20% RH (HC) Duration: 9–10 d. CN measures: Exp1- Before or after HA, Exp2- All before HA		Exp1: ↓T_re(rest)_ after HA ↔ T_re(sleep)_, T_sk(rest)_ and HR_(rest)_ Exp2: ↓T_re(rest)_ (pm) ↔T_sk(rest)_, HR and BP ↓S_(Lat)_ after HA (pm test) ↑S_(Lat)_ (for CN, pm compared to am test) ↓mean T_re_ at S onset (pm) ↔Plasma Na, K, Cl, TP, Alb
Beaudin et al., 2009	HA: 23.78°C, 29.42% RH CN: 24.2°C, 33.5% RH	Number: 12 Sex: male	CH, seated in HC with vapor-impermeable suit (2 h) Conditions: 50°C, 20% RH (HC) Duration: 10 d. Subjects: 12M (8HA, 4CN)		↓T_(es)_ _(rest)_ and mean T_(es)_ threshold for S onset ↔T_sk(rest)_ ↑PV and S (for given T_(es)_)
Fox et al., 1964	HA groups: 38.5°C (CH) Arms were not immersed, plastic bags enclosed both arms	Number: 12 Sex: male Training status: army volunteers	HA groups entered air stream, and sat in hot room with vapor-barrier suits (2 h), with CH and one arm in WT Conditions: 37.9°C (CH and Room), 43°C, 100% RH (air stream), WT temps at 13°C (G1), 37°C (G2), 43°C (G3) Duration: 15 d.		HA groups: ↑Max SC, maintenance of SR, SL (total) and SL (CN arms) ↑SL (arms at 43°C) than CN arms ↔SL of arms at 37°C and CN arms CN Group: ↑SL in treated arm
Henane and Valatx, 1973	Heat sessions had varying stages with maximum temp of 55°C	Number: 9 (no CN) Sex: male Training status: fit, young	HA via 3 h. CH (38°C) heat sessions in HC with 4 stages of different temp (55°C max), WS, WVP and heat loads Duration: 9 d.		↑SC, SO (hourly) and SR (at given T_core_) ↓Time to and T_core_ at S onset ↑HT (core and periphery)
Racinais et al., 2017	HYP, 44–50°C, 50% RH CON, 24°C, 40% RH	Number: 14 Sex: male	HA via rest in HC (1 h) Conditions: 48–50°C, 50% RH Duration: 11 d.		CON: ↓Mean T_re_ HYP: ↑Time to target T_re_ ↑SR ↓Mean HR ↔ TC and TS (CON/HYP)
Pallubinsky et al., 2017	Heat exposure with a temperature ramp 29°C for 60 min 37.5°C for 90 min 26 ± 7% RH	Number: 11 Sex: male Training status: healthy	PMHA via seated rest in HC (4–6 h) Conditions: 33°C, 22% RH Duration: 7 d.		↓T_core_ and Total water loss during temperature ramp ↓Baseline SBP and DBP ↔ Baseline T_core_, basic MR and energy expenditure ↔ Mean T_sk_, HR, SV and CO
Zurawlew et al., 2016	Temperate: 18°C, 40% RH Hot: 33°C, 40% RH	Number: 17 Sex: male Training status: active	Daily HWI (40 min) after 40 min run in temperate conditions Conditions: 18°C (Run), 40°C (HWI) to neck Duration: 6 d.	↑Perf in 33°C 5 km treadmill TT (4.9%) ↔Perf in 18°C 5 km TT	At rest: ↓Mean T_re_ ↑PV During ex: ↓ T_re_, T_sk_ and T_re_ at S onset (18°C, 33°C) ↑SR ↓RPE (18°C, 33°C) ↓PhS and TS (33°C) ↔VO_2_ and RER ↓End ex T_re_ (33°C), HR and RPE (18°C, 33°C)
Zurawlew et al., 2018	33°C, 40% RH	Number: 10 Sex: male	Daily HWI (40 min) after 40 min run in temperate conditions Conditions: 20°C (Run), 40°C (HWI) to neck Duration: 6 d.		↓ T_re(rest)_, T_re_ at S onset, end ex T_re_, T_sk_, end ex HR, TS, mean VO_2_ and RPE ↔RER, WBSR and PV
Brazaitis and Skurvydas, 2010	23°C, 40% RH	Number: 25 Sex: male and female Training status: healthy	Passive lower body heating via 7 HWI (45min to waist) Conditions: 44°C (HWI), 23°C (Air), 40% RH Duration: 14 d.		↓T_re_ (rest/after heat) ↓HR (45 min of heat) ↔HR (pre-heat) ↑RLBM and SR ↓PhS ↔Central/peripheral fatigue_(ex)_
Kanikowska et al., 2012	26°C, 50% RH (HC), rest 30 min then leg HWI (42°C) for 30 min	Number: 6 Sex: male Training status: healthy	CH at 37.5°C, HWI to chest (10 min), HC with blanket (90 min) Conditions: 42°C (HWI), 40°C and 50% RH (HC) Duration: 9 d.		↓HR and T_(tymp)_ at rest ↑SR ↔Plasma cortisol levels ↑POMS score for anger- hostility subscale
Allan and Wilson, 1971	40°C (Bath) maintaining T_(tymp)_ between 37.6 and 38.6°C	Number: 3 (no CN) Sex: NR	Daily HWI (60min) maintaining T_core_ ≥38°C Conditions: 40°C (bath) Duration: 21 d.		↑SR at rest ↓ S_[Na]_
Shin et al., 2013	Not reported	Number: 9 (no CN) Sex: male Training status: healthy	Half body HWI (30 min) for 10 sessions in a HC Conditions: 42 ± 0.5°C (bath), 26 ± 0.5°C, 60 ± 3.0% RH (HC) Duration: 21 d. (alternate)		↓Basal T_(tymp)_ and mean body temp ↑SL
Bonner et al., 1976	Seated rest in 48°C db, 33°C wb, 36% RH (HC) 1.5 m.s^−1^ air movement for 155 min, then cycling at 50 rpm for 30 min	Number: 5 (no CN) Sex: male Training status: healthy	Hot bath in a HC, with CH at 38.5 ± 0.2°C for 60 min Conditions: 41°C (bath), 40°C db 28°C wb (HC) Duration: 13 d. (alternate)		↑SR at rest ↑RLBM ↓T_core_ and HR_(ex)_ ↑PV ↑ plasma [Na]/[K] ratio ↓plasma osmolarity and absolute Hct and PP concentrations ↔Plasma cortisol/aldosterone

*↔, no change; Alb, Albumin; am, morning; BM, body mass; bpm, beats per minute; CH, controlled hyperthermia; CN, control; CON, normothermic state, ; d, day(s) ; db, dry bulb thermometer; ex, exercise; Exp, experiment; HA, heat acclimated group; HC, heat chamber; Hct, haematocrit; HR, heart rate; h, hour(s); HR_ex_, Heart rate during sub-max exercise; HRR_60s_, Heart rate recovery at 60s post-exercise; HT, heat transfer; HWI, hot water immersion; HYP, hyperthermic state; MR, metabolic rate; MVC, maximal voluntary contraction; NR, not reported; Perf, performance; PhS, physiological strain; PhS_(ex)_ , physiological strain during submaximal exercise; pm, afternoon; PMHA, passive mile heat acclimation; POMS, profile of mood states; PP, plasma protein; RCV, red cell volume; RER, respiratory exchange ratio; RH, relative humidity; RLBM, relative loss of body mass; RPE, rating of perceived exertion; RPE_(ex)_ , rating of perceived exertion during submaximal exercise; rpm, revolutions per minute; S, sweat; S_(Lat)_ , sweat latency; S_[Na]_ , sweat Sodium concentration; SC, sweat capacity; SL, sweat loss; SO, sweat output; SR, sweat rate; T_core_, core body temperature; T_(es)_ , esophageal temperature; T_re_, rectal temperature; T_re(ex)_ , rectal temperature during submaximal exercise; T_sk_, skin temperature; T_(tymp)_ , tympanic temperature; TBV, total blood volume; TC, thermal comfort; Temp, temperature; TP, total protein; TS, thermal sensation; TT, time-trial; TTE, time to exhaustion; VO_2_, oxygen consumption; W, Watts; wb, wet bulb thermometer; WBSR, whole body sweat rate; WT, water tank; WVP, water vapor pressure*.

### Protocols

The passive HA strategies used within the literature to date have included resting in a heat chamber (Henane and Valatx, 1973; Beaudin et al., 2009), sauna (Leppaluoto et al., 1986; Scoon et al., 2007; Stanley et al., 2015), or hot bath (Allan and Wilson, 1971; Brazaitis and Skurvydas, 2010; Zurawlew et al., 2016, 2018). Some protocols had included exercise beforehand (Scoon et al., 2007; Stanley et al., 2015; Zurawlew et al., 2016, 2018) and others had not included any exercise (Fox et al., 1964; Allan and Wilson, 1971; Henane and Valatx, 1973; Leppaluoto et al., 1986; Shido et al., 1999; Beaudin et al., 2009; Brazaitis and Skurvydas, 2010; Kanikowska et al., 2012).

Heat chamber protocols ranged from 9–15 days in duration, some of which included the use of vapor impermeable suits (Fox et al., 1964; Beaudin et al., 2009) or blankets (Kanikowska et al., 2012) to reduce sweat evaporation, heat loss and/or promote achievement of a target core temperature. Sauna (i.e., Finnish bath) protocols typically entailed exposure to dry heat (with a relative humidity ranging between 10 and 20%) and high temperatures elicited by an electric heater with hot rocks in a wooden floored and paneled room (Hannuksela and Ellahham, 2001) either once (Stanley et al., 2015), or twice daily (Leppaluoto et al., 1986), by itself (Shido et al., 1999) or following exercise (Scoon et al., 2007), for a period of anywhere between 7 and 21 days. Sauna suits have also been used to replicate sauna conditions (Mee et al., 2017).

Hot-water immersion (HWI) protocols used hot baths to the level of the neck (Allan and Wilson, 1971; Zurawlew et al., 2016, 2018), chest (Kanikowska et al., 2012) or waist (Brazaitis and Skurvydas, 2010) to maintain a specific body temperature typically over a set duration, anywhere from 6 to 21 days, consecutively (Zurawlew et al., 2016, 2018), or on every other day (Brazaitis and Skurvydas, 2010). The HWI's were either carried out following exercise at a controlled workload in a laboratory (temperate conditions) (Zurawlew et al., 2016, 2018), preceding rest in a heat chamber (Kanikowska et al., 2012), or with no other added intervention (Allan and Wilson, 1971; Brazaitis and Skurvydas, 2010; Shin et al., 2013). Water temperatures were controlled by adding or removing water, and when the controlled hyperthermia technique was used, the target core temperature was kept constant by adjusting the degree of immersion in the water (Allan and Wilson, 1971). The duration of heat stimulus per session for the different types of protocols were typically around 2–4 h for heat chambers (Fox et al., 1964; Henane and Valatx, 1973; Shido et al., 1999; Beaudin et al., 2009), 30–60 min for saunas (Leppaluoto et al., 1986; Scoon et al., 2007; Stanley et al., 2015) and around 30–60 min for HWI (Allan and Wilson, 1971; Brazaitis and Skurvydas, 2010; Kanikowska et al., 2012; Zurawlew et al., 2016). The temperatures used were generally the lowest in the HWI protocols (water temp: ~40°C), followed by heat chamber protocols (ambient temp: ~50°C), with the highest ambient temperatures typically being provided by saunas (ambient temp: ~80°C). Following an HA program, newly acclimated individuals typically displayed several performance, perceptual and physiological adaptations.

### Performance Responses

Within the HA literature, performance responses have been measured by an improvement in a progressive exercise test (i.e., VO_2max_ and/or peak power test), exercise duration at a fixed workload (i.e., time to exhaustion), or performance time or power output over a set exercise duration or distance (i.e., time-trial). A 32% improvement in running time to exhaustion (at the runners' best speed over 5 km) was observed after 12 sauna bathing sessions at 90°C for 30 min following exercise training bouts in a cool environment, across a 21-day protocol (Scoon et al., 2007). This was reported to be equivalent to a 1.9% improvement in an endurance-based time trial (Scoon et al., 2007). In contrast, no ergogenic effect was demonstrated in peak power output during a graded cycling test to exhaustion following 10 × 30 min sauna exposures (87°C, 11% RH) following or during the warm down of a training session, despite improved physiological markers of heat adaptation (Stanley et al., 2015). Both protocols used similar duration, number and intensity of sauna exposures following exercise, as well as male subjects with similar training status. However, the differences described by Stanley and colleagues may be explained by the fact that athletes completed the second maximal cycling performance test the week following the monitoring period (~15 days after the sauna period). Some adaptations may have been lost during the period prior to the maximal performance test, since research has highlighted that HA benefits can be lost over time without continued repeated heat exposure, with 1 day of acclimation potentially lost with every 2 days lacking heat stress exposure (Daanen et al., 2018). Furthermore, both studies used seven or less participants, and did not use the same type of performance test.

In the only study performed to date to measure endurance performance in an externally valid time trial performance test, the use of post-exercise HWI to the level of the neck improved endurance performance in a hot environment (Zurawlew et al. (2016). The authors reported a 4.9% improvement in a 5 km treadmill time-trial test conducted in hot (33°C) conditions after a 6-day protocol consisting of daily 40 min HWI exposures at 40°C following training, which comprised of 40 min of treadmill running at 65% VO_2max_ in temperate (18°C) conditions. Interestingly, the ergogenic effect was not transferred to performance of the same test under cool conditions (18°C). The notion that HA performance benefits (achieved via active/passive interventions) can be observed in temperate/cool conditions remains debatable (Minson and Cotter, 2016). Active-HA based research has indicted that performance benefits following acclimation/ acclimatization interventions may be elicited in cool/temperate conditions (Lorenzo et al., 2010), while others reported contradictory findings (Karlsen et al., 2015). Importantly, there seems to be no clear evidence that HA could be detrimental to performance in cool environments, thus HA strategies are still recommended when anticipated environmental conditions for competition are hot/unknown (Minson and Cotter, 2016).

Most of the studies included in this review focused on physiological responses to HA, as presented below, while only three studies measured endurance performance following passive HA protocols. Such research suggests performance can be significantly improved. Future studies could include more performance tests, specifically time trials, in order to provide a clearer consensus regarding the performance benefits of such interventions.

### Physiological Responses

Changes in several physiological variables have been identified following HA protocols. These have included a reduction in metabolic rate, heightened skin blood flow and sweat responses, increased plasma volume, improved cardiovascular stability, and fluid balance and improved thermal tolerance, which are all contributors to the benefits of HA (Périard et al., 2015). Furthermore, the physiological responses that typically occur earliest are reductions in heart rate, skin and core temperature, as well as a rise in sweat rate and work capacity, generally occurring within the first week of HA, following which other cardiovascular and sudomotor adaptations are likely to occur (Racinais et al., 2015). The thermoregulatory, cardiovascular and other physiological alterations that have occurred following passive HA will be discussed in the following sections.

#### Temperature Responses

Increased core and/or skin temperatures that accompany exercise in hot conditions can indirectly reduce maximal oxygen uptake and thus negatively influence aerobic performance (Cheuvront et al., 2010). Core temperature responses when humans are exposed to hot environmental temperatures are influenced by hydration status, if hypo-hydration accompanies compensatory increase of sweat outputs, elevations in core temperature and heat storage generally occur in consequence (Kenney et al., 2004). Heat adaptation creates a shift in the “critical environment” (a range of environmental conditions beyond which core temperature cannot be maintained within the normal range), improving the ability to effectively withstand hot environmental temperatures (Kenney et al., 2004). Importantly, effective passive HA interventions have reduced core temperature at rest, during exercise and upon cessation of exercise (Bonner et al., 1976; Leppaluoto et al., 1986; Shido et al., 1999; Zurawlew et al., 2016, 2018). Reductions in resting core (esophageal/ rectal) temperature (~0.26°C reduction on average), have been noted following 10–11 day passive HA regimes that used 1-h, 2-h, or 4-h heat exposures per day. These involved rest in a heat chamber either with (Beaudin et al., 2009), or without (Shido et al., 1999; Racinais et al., 2017) the application of vapor impermeable suits, respectively, as well as a 7-day protocol involving 1-h exposures twice a day to dry heat in a Finnish sauna (Leppaluoto et al., 1986) and HWI with/without exercise beforehand (Bonner et al., 1976; Brazaitis and Skurvydas, 2010; Shin et al., 2013; Zurawlew et al., 2016, 2018). Similarly, the time to reach a specific target core temperature has been increased (~9 min increase) following an 11-day heat chamber protocol using 1-h per day passive heat exposures (Racinais et al., 2017).

The main stimulus for individuals to achieve a lower resting core temperature, and thus heat adaptation, is repeated episodes of elevated core temperature, suggesting that repeated physical exercise in the heat may not be necessary for HA (Regan et al., 1996; Brazaitis and Skurvydas, 2010). Furthermore, simply subjecting individuals to significant heat stress can improve tolerance to greater doses of heat stress by stimulating heat shock protein production and enabling greater cell protection against the adverse effects of high temperatures (Lim et al., 2008). Passively induced HA can also reduce the end-exercise core temperature significantly, as demonstrated by two 6-day interventions involving repeated 40 min HWI at 40°C following running in temperate (18°C and 20°C) conditions on consecutive days, which lead to lower final rectal temperatures in both hot (33°C) (~0.36°C mean reduction), and temperate (18°C) (~0.28°C mean reduction) environments, at the end of treadmill running (Zurawlew et al., 2016, 2018). Such reductions in core temperature can be attributed to a reduced heat storage resulting from improved heat loss mechanisms (Buono et al., 1998) which typically accompany HA, as shown by Henane and Valatx (1973) who reported a heightened heat transfer between the core and periphery, as well as improved sweat responses during 3 h of controlled hyperthermia in a heat chamber (refer to Table 1).

Lower core temperatures have also accompanied exposure to 27°C and 50% relative humidity in a heat chamber at the same time of previous heat exposure, suggesting a timed memory of heat exposure following a passive HA intervention (Shido et al., 1999). These were observed in acclimated individuals who followed a protocol that involved rest in a heat chamber at 46°C, with 20% relative humidity for 4 h on 9–10 consecutive days, to analyze how the thermoregulatory responses differed between the time period when the individuals were exposed to heat stress and the rest of the day. In contrast, a recent study that used post-exercise HWI conducted in the morning, advanced such findings and reported the presence of physiological adaptions gained from the intervention (resting and during exercise in the heat) in both the morning and mid-afternoon (Zurawlew et al., 2018). Additive research is required to provide more robust conclusions with regards to whether HA adaptations (physiological/perceptual) are more apparent at the time of dosage of heat stress in an HA intervention, or if they can be observed similarly throughout the day after HA is complete.

Skin temperature is influenced by environmental temperatures as well as thermoregulatory responses (Cheuvront et al., 2010). Passive HA can reduce skin temperature during submaximal exercise in both hot and cool conditions, as demonstrated by a 6-day post exercise HWI interventions (Zurawlew et al., 2016, 2018). This adaptation could have high relevance for athletes, since previous research has identified that high skin temperatures that accompany exercise in hot conditions, without the presence of considerably high core temperatures, are associated with earlier exercise cessation at light/moderate exercise intensities (Cheuvront et al., 2010). The mechanism of how high skin temperatures contribute to compromised exercise performance can be explained by the fact that the skin blood flow is increased in an attempt to dissipate heat, contributing to the distribution of blood to the periphery, thereby reducing cardiac filling and cardiac output, which in turn contributes to a reduced maximal oxygen uptake (Cheuvront et al., 2010). Additionally, skin temperature influences perceptual comfort, which could impact pacing strategies and performance (Bulcao et al., 2000). With regards to resting skin temperatures, three studies using an either 7 or 10-day protocols with 20 or 26% relative humidity in a heat chamber, with (ambient temperature at 50°C) or without vapor-impermeable suits (ambient temperature at 37.5 or 46°C), reported no significant changes in skin temperatures that were measured post-acclimation from the resting skin temperatures measured prior to the acclimation (Shido et al., 1999; Beaudin et al., 2009; Pallubinsky et al., 2017).

#### Sweat Responses

Evaporative heat loss is the primary means of heat dissipation from the body during physical activity accompanied by significant environmental heat stress, accounting for almost 80% of heat loss, highlighting the importance of the ability to sweat under such conditions to enable adequate thermoregulation (Kenney et al., 2004; Lim et al., 2008). As such, enhanced sweating responses are important physiological adaptations acquired from HA, since sweat evaporation aids the maintenance of thermal balance and the ability to avoid thermal overload (Henane and Valatx, 1973). The time to sweat onset (sweat latency) during resting exposure to heat has decreased (~4 min) following protocols involving seated rest in a heat chamber for 9–10 days (Henane and Valatx, 1973; Shido et al., 1999). Furthermore, sweat onset has commenced at lower associated core temperature following heat chamber protocols at rest (~0.31°C mean reduction) (Henane and Valatx, 1973; Beaudin et al., 2009), and also following HWI protocols (Zurawlew et al., 2016, 2018), where the response has been noted following HA within performance tests conducted in both temperate and hot conditions (Zurawlew et al., 2016). Earlier sweat onset may be attributed to a number of factors including greater thermal input, enhanced sensitivity of the central nervous system, higher skin temperatures at measurement sites, and/or enhanced sensitivity of the sweat gland to a given level of central drive (Shido et al., 1999).

Enhanced sweating capacities, demonstrated by increased sweat outputs and sweat rates, have also been observed following various passive HA protocols. Increased sweat outputs have accompanied controlled hyperthermia heat chamber protocols either with (Fox et al., 1964; Beaudin et al., 2009) or without (Henane and Valatx, 1973) the application of rain/vapor-barrier suits. Additively, enhanced sweat rate responses have accompanied heat chamber protocols that entailed exposure to incremental heat stress (Henane and Valatx, 1973; Racinais et al., 2017) or application of a vapor impermeable suit (Fox et al., 1964), as well as HWI protocols using either daily HWI following exercise (Zurawlew et al., 2016), HWI prior to passive heating in a climate chamber (Kanikowska et al., 2012), or the controlled hyperthermia technique without exercise (Allan and Wilson, 1971; Bonner et al., 1976). The enhanced sweat rate responses following acclimation protocols have occurred at rest upon exposure to heat (Allan and Wilson, 1971; Henane and Valatx, 1973), as well as during exercise in hot and temperate conditions (Zurawlew et al., 2016). Furthermore, enhanced body fluid loss as demonstrated by larger relative reductions in body mass and an increase in whole body sweat loss have accompanied alternate day HWI protocols (Bonner et al., 1976; Brazaitis and Skurvydas, 2010; Shin et al., 2013). Enhanced sweat loss can be beneficial for athletes, provided that it is further accompanied by increases in heat loss via evaporative mechanisms and that athletes maintain adequate hydration in order to prevent a rise in thermal strain, since increased fluid loss from the body can result in progressive dehydration and cardiovascular strain, and a prolonged dehydration can reduce performance and be harmful to health (Kenney et al., 2004; Tyler et al., 2016).

Consequently, the requirement for fluid replacement is greater once heat adaptations have been achieved, due to the enhanced sweat responses. Dehydration has a significant influence on sweat latency and also results in core temperature responses that are the same as if acclimation had not been achieved, further highlighting the importance of ensuring proper hydration status (Shido et al., 1999; Wendt et al., 2007). However, heat-adapted individuals are generally better at maintaining adequate hydration status via improved fluid replacement, noted by reduced voluntary dehydration and improved relationships between thirst and body water requirements in these individuals (Périard et al., 2016). More dilute sweat, potentially achieved via increased reabsorption of electrolytes, can evaporate easier from the skin and is therefore a beneficial response accompanying HA (Tyler et al., 2016). As such, sweat concentration has been reduced after passive HA, as demonstrated by decreased sweat sodium content over a variety of sweat rates following controlled hyperthermia using 40°C HWI (Allan and Wilson, 1971). This, along with a reduction in time to sweat onset, is a common feature of HA (Allan and Wilson, 1971; Henane and Valatx, 1973; Leppaluoto et al., 1986).

#### Hematological Responses

The expansion of plasma volume (hypervolemia) has been induced in men of various training statuses (healthy, active, well-trained and competitive) following heat chamber, sauna and HWI protocols with 40–120 min heat exposures (~11.08% mean increase) (Bonner et al., 1976; Scoon et al., 2007; Beaudin et al., 2009; Stanley et al., 2015; Zurawlew et al., 2016). Importantly, plasma volume expansion can improve maximal oxygen uptake (Davies, 1979) and contribute to improved cardiovascular stability, by allowing for a reduction in heart rate (via contribution to an improved distribution of blood flow during exercise and improved ventricular filling) and an increase in stroke volume (Wendt et al., 2007; Racinais et al., 2015; Périard et al., 2016). Plasma volume expansion may also contribute to improved heat transfer between exercising muscle and skin by raising the specific heat capacity of the blood (Casa, 2018). Reductions in serum potassium, sodium and iron levels have been reported after a sauna bathing protocol involving rest for an hour, twice daily in 80°C conditions (Leppaluoto et al., 1986); as well as reduced plasma osmolarity, plasma protein concentration and absolute values of haematocrit, with no significant changes in plasma cortisol or aldosterone levels after a protocol involving 1-h of HWI (41°C) for 13 days (Bonner et al., 1976). However, no significant changes in plasma sodium, potassium, total protein, and albumin levels were noted after a heat chamber involving rest for 4 h at 46°C and 20% relative humidity (Shido et al., 1999). Both protocols used healthy men and did not include exercise, but made use of different heat stimuli temperatures, duration and methods that could potentially explain the conflicting observations. Other cardiovascular adaptions such as increased red cell volume, body mass and total blood volume have accompanied post exercise sauna bathing in long distance runners (Scoon et al., 2007). Despite limited information regarding skin blood flow changes with passive HA interventions, there is general consensus that following HA maximal skin blood flow is unchanged (Périard et al., 2015). However, skin blood flow responses may improve (redistribution and reduced requirements) as a result of enhanced sweating capacity and thus reduced skin temperature responses (Schmit et al., 2018). Additively, the expansion of blood volume allows more blood to be pumped, as required, to muscles, organs, and the skin. Evidently, the cardiovascular adaptations acquired following heat acclimation contribute to improved cardiovascular stability accompanying physical exercise under hot conditions (Garrett et al., 2011).

#### Heart Rate Responses

A recent systematic review on the effects of active HA on physiology, perception and exercise performance in the heat stipulated a lowered heart rate during exercise, but less of an impact on reducing resting values (Tyler et al., 2016). In opposition, the passive HA protocols explored in this review have noted significant changes in resting heart rate values (Leppaluoto et al., 1986; Kanikowska et al., 2012; Racinais et al., 2017). Sauna bathing for 30 min following training lead to trivial-to-moderate reductions in heart rate upon waking, but no change in the peak heart rate values for well-trained cyclists (Stanley et al., 2015); and similarly, Finnish sauna dry heat exposure for 1-h, twice per day (Leppaluoto et al., 1986), HWI either following exercise (Zurawlew et al., 2016, 2018), or preceding rest with a blanket in a heat chamber (Kanikowska et al., 2012), and passive heat exposure using a heat chamber (Racinais et al., 2017) have all successfully lowered resting heart rate values. Similarly, following a HA protocol that made use of passive lower body heating achieved by HWI, the reading for heart rate after 45 min of passive heating had decreased (Brazaitis and Skurvydas, 2010), demonstrating successful HA in the participating individuals. In contrast, no significant changes in resting heart rate values were noted after a 7 or 9-day interventions where individuals rested in a heat chamber for 4–6 h per day (Shido et al., 1999; Pallubinsky et al., 2017).

Exercising heart rate values have also shown a reduction after HWI protocols (Bonner et al., 1976; Kanikowska et al., 2012; Zurawlew et al., 2016, 2018). Importantly, only four studies used exercise tests, with only two that measured end-exercise heart rate values (Zurawlew et al., 2016, 2018), and one that measured heart rate during submaximal exercise (Stanley et al., 2015), but this study reported equipment malfunction and loss of some heart rate related data (0–13%). Consequently, there is limited information on the exercising heart rate responses during performance testing after passive HA protocols.

#### Thermoregulatory Strain Responses

Thermoregulatory strain can be calculated using rectal temperature and heart rate during submaximal exercise where the information is converted to a scale ranging from 0 to 10. Reduced physiological strain indexes (Moran et al., 1998) in hot conditions have been reported after HWI of the lower body (Brazaitis and Skurvydas, 2010), and HWI following exercise in temperate conditions (Zurawlew et al., 2016). Such changes may decrease negative contributions to performance including lowered muscle recruitment, excess fluid loss from the body, feelings of discomfort and a disturbed relationship between oxygen consumption and heart rate (Tyler et al., 2016).

### Perceptual Responses

Hyperthermia and heat stress contribute to the increased perception of exertion during submaximal exercise in hot ambient conditions, highlighting the benefit of improved ability of the human body to dissipate heat following HA for athletes (Pandolf, 2001; Armada-da-Silva et al., 2004; Cheuvront et al., 2010). The implications for athletes of a higher perceived exertion as a result of increased heat stress is that it may influence other factors associated with exercise performance, such as behavior and motor neural firing that is driven by motivation (Cheuvront et al., 2010). Consequently, perception of heat can contribute to premature fatigue and negatively influence performance (Tyler et al., 2016; Stevens et al., 2018), and as such, athletes may perform better with improved perception of thermal load (Chalmers et al., 2014).

There are a number of perceptual responses that have been examined in the literature, for example, mood (Kanikowska et al., 2012), thermal sensation (Young et al., 1987; Schulze et al., 2015; Zurawlew et al., 2016; Racinais et al., 2017), thermal comfort (Young et al., 1987; Bulcao et al., 2000; Schulze et al., 2015; Kelly et al., 2016; Racinais et al., 2017), and perceived exertion (Zurawlew et al., 2016). Some of these studies investigated the effect of HA on perceptual responses (i.e., POMS, thermal sensation, thermal comfort and perceived exertion) (Kanikowska et al., 2012; Kelly et al., 2016; Zurawlew et al., 2016; Racinais et al., 2017). Others investigated perceptual responses during heat stress tests (Young et al., 1987; Bulcao et al., 2000; Schulze et al., 2015).

Thermal sensation refers to the recognition of how hot or cold the body feels, whereas thermal comfort is a measure of how the individual interprets their thermal state, and could have a larger influence on exercise regulation in the heat than the actual thermal state, since it is thought to be the initiator of behavioral thermoregulation (Bulcao et al., 2000; Schulze et al., 2015; Mee et al., 2017). Thermal comfort in hot environments can be improved with the adaptations gained following repeated exposure to hot conditions (Périard et al., 2016). Different scales have been used to assess thermal sensation and thermal comfort in previous research, with some scales not clearly distinguishing between the two variables (Schweiker et al., 2017). The use of a seven-point ASHRAE scale to measure thermal sensation, and a different four-point scale to assess thermal comfort was proposed by Gagge et al. (1967). Interventions using HWI have lowered ratings of perceived exertion during and after exercise in both hot (33°C) and temperate (18°C or 20°C) conditions for participants who followed a 6-day protocols (Zurawlew et al., 2016, 2018). In the same studies, reductions in thermal sensation were observed during exercise in the hot conditions, and at the end of exercise in temperate conditions, respectively (Zurawlew et al., 2016, 2018). In contrast, another recent study reported no changes in either thermal sensation or comfort measures reported by male participants after an 11-day heat chamber protocol (Racinais et al., 2017). Improvements in perceptual responses following HA protocols may allow up-regulated self-paced exercise, and therefore are worth investigating further. Kanikowska et al. (2012) made use of a Japanese version of the profile of mood states questionnaire at test sessions to analyse feelings during a HWI followed by blanket resting in a heat chamber protocol, and reported higher scores on the anger-hostility subscale from the six healthy male participants, indicating mood decrements after HA.

## Practical Applications and Recommendations for Athletes

Based on the research described herein, it is evident that passive strategies using hot rooms/HWI can be employed to induce heat adaptations, as per measured improvements in performance, thermoregulatory, cardiovascular, and perceptual responses. Exercise is not necessary for the physiological adaptations to occur following passive protocols, however including exercise under temperate ambient conditions in the regime may lead to larger thermoregulatory-adaptive responses than application of heat stress alone, since it can elicit larger rises in core temperature, a major contributing factor to heat adaptation (Casadio et al., 2016). Additively, combining exercise and passive heating in the intervention may induce greater heat shock protein synthesis than utilizing either method alone (Casa, 2018). Importantly, research has highlighted that the magnitude of heat adaptations can depend on the method of induction, the initial heat exposure status and also the number, duration and frequency of heat exposures involved in the HA protocol (Périard et al., 2016; Tyler et al., 2016). Research has also indicated that individual training status could influence the magnitude and timeframe of acclimation in response to HA interventions, (Sawka et al., 2015; Daanen et al., 2018) although information regarding passive HA is limited. The rationale for this suggestion is that well-trained individuals may naturally be partially heat adapted, thus acclimate faster, as they are likely to already have higher aerobic capacity than untrained individuals. In contrast, a recent study concluded that the inter-individual adaptive responses to a 10-day active HA intervention was not related to baseline maximal oxygen uptake (Corbett et al., 2018), as such we cannot conclude whether untrained individuals may experience greater HA benefits in comparison to trained individuals.

The application of post-exercise heat stress can induce numerous physiological adaptions and may improve endurance performance (Scoon et al., 2007; Stanley et al., 2015; Zurawlew et al., 2016). It is important to note that such performance improvements have only been demonstrated under hot but not temperate conditions (Zurawlew et al., 2016). For these passive protocols which used post-exercise application of heat stress, exercise in temperate conditions has typically been performed immediately before entering a sauna or bath. Importantly, while passive strategies may be logistically easier to incorporate into complex training programs and schedules of elite athletes, passive heat exposures alone may not be as effective for the development of sport-specific adaptations in comparison to active/exercise-induced HA (Daanen et al., 2018). Based on the information gathered from this review, for successful passive HA, the heat stimulus should ideally have a minimum duration of 30 min per session, employed on consecutive days, when possible, and the time of day for heat application may not be important for adaptation. Furthermore, the ambient temperatures that have been used are at least 80°C for sauna, 45–50°C for heat chambers and around 40°C for HWI (see Table 1). If the controlled hyperthermia technique is used, the target core temperature should be around 38.5°C, which has been shown to be effective (Patterson et al., 2004). Furthermore, other research has suggested that elevating core temperature beyond 38.5 degrees during HA is not necessary, as it does not further enhance the acclimation benefits (Gibson et al., 2015). Only 6–7 exposures are required to induce beneficial physiological and performance responses (Brazaitis and Skurvydas, 2010; Zurawlew et al., 2016) but most protocols have used the medium term duration of 8–14 days (Scoon et al., 2007; Beaudin et al., 2009; Zurawlew et al., 2016). Despite the fact that most complete adaptations accompany long term interventions, thus may be best practice for ultra-endurance events (Périard et al., 2015), successful medium term protocols may be more favorable for endurance athletes, since they are slightly less time-consuming and can induce complete thermoregulatory benefits as well as more extensive protective effects than short term protocols (Casadio et al., 2016). Current recommendations state that heat acclimation protocols should be conducted immediately prior to competition in order to limit the decay of adaptations (Casa, 2018). Passive heating could also be combined with active HA protocols when time is limiting and active HA is possible, as demonstrated by Mee et al. (2017), who reported accelerated thermal, cardiovascular and perceptual adaptations in females who were passively heated prior training in the heat.

Use of HWI protocols for endurance athletes who train in a cool environment may offer the most practical benefits, since they do not demand lengthy heat exposures (as required by heat chamber protocols), or extremely high ambient temperatures (as are the nature of sauna protocols). Furthermore, hot baths following physical training in temperate conditions can successfully raise core and skin temperatures necessary for heat adaptations to be initiated, and have improved exercise performance, highlighting the relevance for athletes. Despite clear consensus as to whether most of the resting and exercising physiological adaptations acquired could be transferred to performance in a cool environment, there is no evidence to suggest that HA interventions are detrimental to subsequent performance in a cool environment (Minson and Cotter, 2016). While we can suggest that athletes should commence HWI immediately after exercise, it is unclear how long the window is between finishing a session and commencing HWI to obtain the additional benefit. Most studies of this nature commenced heating immediately following the exercise (Scoon et al., 2007; Stanley et al., 2015; Zurawlew et al., 2016).

In order to improve safety when utilizing such protocols, consideration should be geared toward the timing of induction and the environmental/ambient conditions, with pre-disposing factors for heat illness having been identified before implementation of any kind of HA intervention (Casa, 2018). Individuals must keep in mind that heat transfer/ dissipation is also influenced by the surface area of the body, with faster rates of heat dissipation generally occurring in conjunction with larger body surface area to volume ratios (West et al., 2015). Other research has noted that lower absolute metabolic heat production occurs in runners of lower body mass compared to heavier individuals, therefore coaches must monitor heavier athletes during the HA process, as these individuals may be at increased risk of heat illness (Dennis and Noakes, 1999). Importantly, heating sessions should not be undertaken by athletes who have acute injuries, oedema, vascular disease, wounds, or infections (Wilcock et al., 2006). Notably, when water temperatures are higher than core temperatures, the thermal gradient changes from external to internal, leading to progressive heat influx which could contribute to hyperthermia (Taylor, 2006). However, if conducted in a controlled manner, the resultant heat influx can be beneficial, since repeated episodes of elevated core temperature are necessary for heat adaptation (Brazaitis and Skurvydas, 2010). Some successful HWI interventions have even made use of water temperatures above 40°C (Allan and Wilson, 1971; Bonner et al., 1976; Brazaitis and Skurvydas, 2010; Kanikowska et al., 2012; Shin et al., 2013; Zurawlew et al., 2016, 2018). Naturally, it is important to consider that when submerged in water with a temperature greater than that of body temperature, avenues for heat loss via sweating are limited, and consequently, the body will progressively gain heat. Hence, HWI (especially after exercise) could potentially cause heat illness and therefore important safety measures should be implemented. Such safety measures should include using thermal scales to monitor perceptions of hotness, continuous measurement of water temperatures and/or supervision of immersions and monitoring the signs of hyperthermia. Furthermore, drinking water/fluids must be available before, during and after heat sessions, and athletes should discontinue heat sessions accompanied by signs of heat illness, light-headedness, nausea or extreme discomfort (Casa, 2018). Finally, most athletes are likely to have access to a bath in their own home, and as such, employing the HWI strategy may work in favor of time, convenience, comfort and cost, enabling use in a practical setting.

## Conclusion and Future Research Directions

Extensive research has been conducted on HA, with the majority geared toward active rather than passive strategies. Passive strategies can be used to induce heat adaptation, as measured by improvements in endurance capacity and performance, reduced resting and exercising core temperature, lower skin temperature at sweat onset, enhanced sweat capacity, expansion of plasma volume, reduced resting, and exercising heart rate, as well as lowered physiological strain index, rating of perceived exertion and thermal sensation (Shin et al., 2013; Casadio et al., 2016; Tyler et al., 2016). Such changes have been noted in participants of different sex, training status, and sport disciplines. Passive heat stress can be provided by saunas, HWI or environmental chambers. If possible, exercise bouts should be employed prior to heat sessions, rather than passive heating alone, with minimal delay between training and the application of heat stress, for optimal thermoregulatory-adaptive response. Heating bouts should have a minimum duration of 30 min per session and be employed on consecutive days, when possible with interventions having a minimum of 6–7 exposures for successful adaptations.

Passive HA strategies may be more practical than active strategies for athletes training in winter months, residing in cool environments, or with financial constraints (Casadio et al., 2016). The passive strategies that have been explored to date have focused heavily on the physiological responses acquired, while few studies have measured changes in exercise performance which is highly relevant to athletes and practitioners (Wilcock et al., 2006). Additional investigations are needed to provide information regarding the magnitude of performance changes that can occur following passive HA interventions by testing trained athletes in ecologically valid performance tests (i.e., time-trial protocols). Different time intervals, and comparisons between effects of pre- as opposed to post-exercise application of heat stress should also be explored in order to provide more robust recommendations for athletes using these strategies, who likely have delays between completing a training session and commencing HWI for several logistical reasons. Furthermore, future research could examine the combination of active and passive protocols (i.e., passive heat exposure following exercise in the heat) to investigate if this double heat training technique could provide a further boost to the performance, physiological and perceptual adaptations. Additively, research directed toward heat adaptation with consideration for sport specificity is warranted (i.e., protocols tailored to the specific demands of the chosen sport). The review has also identified that perceptual responses to HA achieved via protocols that involve rest in environmental chambers/saunas have not been explored thoroughly within the literature to date. This has also been highlighted by Tyler et al. (2016), who found limited data with regards to perceptual measures, including ratings of perceived exertion and thermal sensation, accompanying HA studies in a recent systematic review. As such, future research should employ more perceptual measurements accompanying HA strategies for athletes, since reducing the perception of heat stress may delay the onset of fatigue during exercise performance (Armada-da-Silva et al., 2004). This information could help lower the incidence of heat stress and improve performance for individuals competing in the heat.

## Author Contributions

All authors collaboratively developed the concept of the review and the inclusion criteria. SH drafted and finalized the manuscript, PH, SZ, and CS reviewed the drafts and provided critical feedback.

### Conflict of Interest Statement

The authors declare that the research was conducted in the absence of any commercial or financial relationships that could be construed as a potential conflict of interest.
